# Southern Medical College

**Published:** 1884-07-20

**Authors:** 


### *SOUTHERN MEDICAL COLLEGE.*

We invite attention to the advertisement of the above school in this issue. The SOUTHERN MEDICAL COLLEGE is now more fully and thoroughly equipped for teaching than at any previous session. The Ivy Street Hospital, which is on a lot adjacent to the College, is in full and successful operation, and will give peculiar advantages to the students of the SOUTHERN MEDICAL COLLEGE. There are indications of a large class at the approaching session, which will open on the 7th of October uext.

